# Understanding and Mimicking Glioblastoma Cells Migration Strategies Through Hydrogel-Based 3D Models

**DOI:** 10.3390/biology15211987

**Published:** 2026-11-03

**Authors:** Alessandro Lo Giudice, Martina Di Marco, Giulia Maria Giorgia Lo Piano, Francesca Chiara Cecala, Claudia Campanella, Francesco Cappello, Celeste Caruso Bavisotto, Giuseppa D’Amico, Alessandra Maria Vitale

**Affiliations:** 1 Department of Biomedicine, Neuroscience and Advanced Diagnostics, University of Palermo, 90127 Palermo, Italy; alessandro.logiudice03@unipa.it (A.L.G.); martina.dimarco@unipa.it (M.D.M.); giuliamariagiorgia.lopiano@unipa.it (G.M.G.L.P.); francescachiara.cecala@you.unipa.it (F.C.C.); claudia.campanella@unipa.it (C.C.); francesco.cappello@unipa.it (F.C.); giuseppa.damico01@unipa.it (G.D.); alessandramaria.vitale@unipa.it (A.M.V.); 2 Mitchell Center for Neurodegenerative Diseases, Department of Neurology, University of Texas Medical Branch (UTMB), Galveston, TX 77555, USA; 3 Euro-Mediterranean Institute of Science and Technology (IEMEST), 90139 Palermo, Italy

**Keywords:** GBM, tumor invasion, tumor migration, oncostream, 3D hydrogel

## Abstract

Glioblastoma (GBM) is one of the most aggressive primary brain tumors in adults, characterized by rapid growth, diffuse infiltration of surrounding brain parenchyma, and resistance to conventional therapies, resulting in poor prognosis. The diffuse infiltrative behavior of GBM represents a major therapeutic challenge, as cancer cells can disseminate beyond identifiable margins of the tumor, escaping complete eradication and contributing to recurrence. GBM cell migration is a heterogeneous process that can occur through distinct strategies, including single-cell and collective patterns, as well as through the formation of multicellular structures known as “oncostreams”. Understanding the mechanisms underlying these migratory modalities may help identify new targets for limiting local tumor dissemination and disease progression. In this context, a variety of in vitro models have been developed to investigate these processes. Among these, hydrogel-based scaffolds represent a promising platform to recapitulate some selected biochemical and biophysical properties of the brain extracellular matrix, enabling the investigation of how the extracellular environment cues influence GBM cell migration and invasion. In this review, we discuss the major cellular strategies of GBM cell migration and tissue invasion and critically examine hydrogel-based in vitro models used to investigate these processes, highlighting their design principles, biological relevance, and current limitations.

## Simple Summary

Glioblastoma (GBM) is an aggressive brain tumor that is difficult to treat because its cells can spread into the surrounding brain parenchyma, making it almost impossible to remove the entire tumor through surgery. Tumor cells can move in different ways, individually or together, or by forming complex, organized groups that help them invade surrounding tissue. Understanding how GBM cells migrate and how the environment around them influences this process may help researchers find new ways to limit tumor spread and progression. This review describes the major cellular strategies of GBM cell migration and tissue invasion, and examines some of the most innovative three-dimensional (3D) hydrogel-based models currently used to study these processes. These systems can reproduce selected features of the environment in which tumor cells grow and spread. They also allow researchers to investigate how changes in the surrounding tissue affect cell movement and to test potential treatments under more realistic conditions. Although these models cannot yet fully reproduce the complexity of a human brain, they offer valuable opportunities to understand how GBM spreads, identify new molecular targets, and support the development of future therapeutic strategies.

## 1. Introduction

Glioblastoma (GBM) is one of the most aggressive primary brain tumors, classified as an IDH-wildtype diffuse astrocytic glioma, CNS WHO grade 4, based on specific histological and/or molecular features [1]. Its aggressive clinical course is associated with extensive intratumoral heterogeneity, rapid proliferation, necrosis, microvascular proliferation, a highly invasive nature, and resistance to conventional therapies, collectively contributing to poor prognosis [2].

One of the major challenges in GBM treatment is the diffuse, infiltrative behavior of tumor cells, which can spread far beyond the radiologically visible tumor margins, preventing complete surgical resection and contributing to disease recurrence, even following multimodal treatment [3]. Therefore, understanding the biological mechanisms underlying GBM cell migration and invasion abilities is crucial for identifying novel therapeutic targets and developing strategies to limit local tumor dissemination and delay tumor progression. Importantly, migration and invasion describe the same fundamental process, i.e., the movement of cells from one location to another, occurring under different conditions. While ‘migration’ denotes the general movement of cells in response to chemical or mechanical signals, ‘invasion’ refers to the process by which cells infiltrate or traverse surrounding tissue or extracellular barriers through mechanisms that may either involve or be independent of proteolytic activity and matrix remodeling.

GBM cells do not rely on a single, fixed mode of migration, but they exhibit high plasticity, adapting their behavior to the biochemical and physical properties of the surrounding microenvironment. Particularly, their migratory behavior is strongly influenced by interactions with the surrounding extracellular matrix (ECM) and non-neoplastic components of the brain [4]. Therefore, investigating these processes requires experimental platforms able to reproduce key features of the tumor microenvironment (TME) and to recapitulate the complex interaction between tumor cells and extracellular components.

In this context, hydrogel-based three-dimensional (3D) scaffolds have emerged as promising in vitro models because their composition, architecture, and mechanical properties can be tailored to investigate how ECM-related cues influence GBM cell migration and invasion, providing a more physiologically relevant environment than conventional two-dimensional cultures [5].

Accordingly, this review aims to summarize the molecular and cellular mechanisms driving GBM cell migration and invasion and to discuss current in vitro models that enable the investigation of these complex processes to ultimately find new strategies to identify molecular and biomechanical targets for limiting tumor invasion and progression.

### Literature Search Strategy

The present article is a narrative review focused on describing the cellular and molecular mechanisms involved in the migration and invasion of GBM cells, mainly focusing on single-cell and collective migration, oncostream formation, and the use of hydrogel-based 3D systems for studying these processes. The literature search for this review article was conducted on PubMed and Google Scholar databases between July and August 2026, with the last search being carried out in August 2026. In order to identify the most relevant literature regarding this subject, the search terms used were based on the topics discussed in this article and included the following keywords: “oncostreams”, “glioblastoma invasion”, “glioblastoma migration”, “hydrogel”, “collagen”, “3D matrix” and “hyaluronic acid”. The studies conducted after 2020 had priority due to their current importance for the field, while studies performed prior to this date were considered in case they addressed fundamental concepts or biological principles.

## 2. From Single-Cell Migration to Self-Organized Multicellular Structures

One of the greatest challenges in the treatment of GBM is its remarkable ability to infiltrate the surrounding brain parenchyma, as infiltrating cells disseminate far beyond the visible tumor margins, escaping complete surgical resection, surviving conventional therapies, and ultimately driving tumor recurrence [6]. Increasing evidence indicates that GBM cell migration is a highly dynamic process sustained by remarkable cellular plasticity and multiple distinct strategies [7]. Although significant progress has been made in understanding the clinical and biological behavior of GBM, the mechanisms underlying tumor cell migration patterns remain poorly understood. Throughout this section, we will examine the various modes of cell migration observed in malignant gliomas, focusing on GBM, from single-cell to collective patterns, and, ultimately, the formation of multicellular structures known as oncostreams, discussing their similarities and differences in comparison to the well-established migratory patterns described so far.

### 2.1. Single Cell Migration Pattern

**Figure 1:**
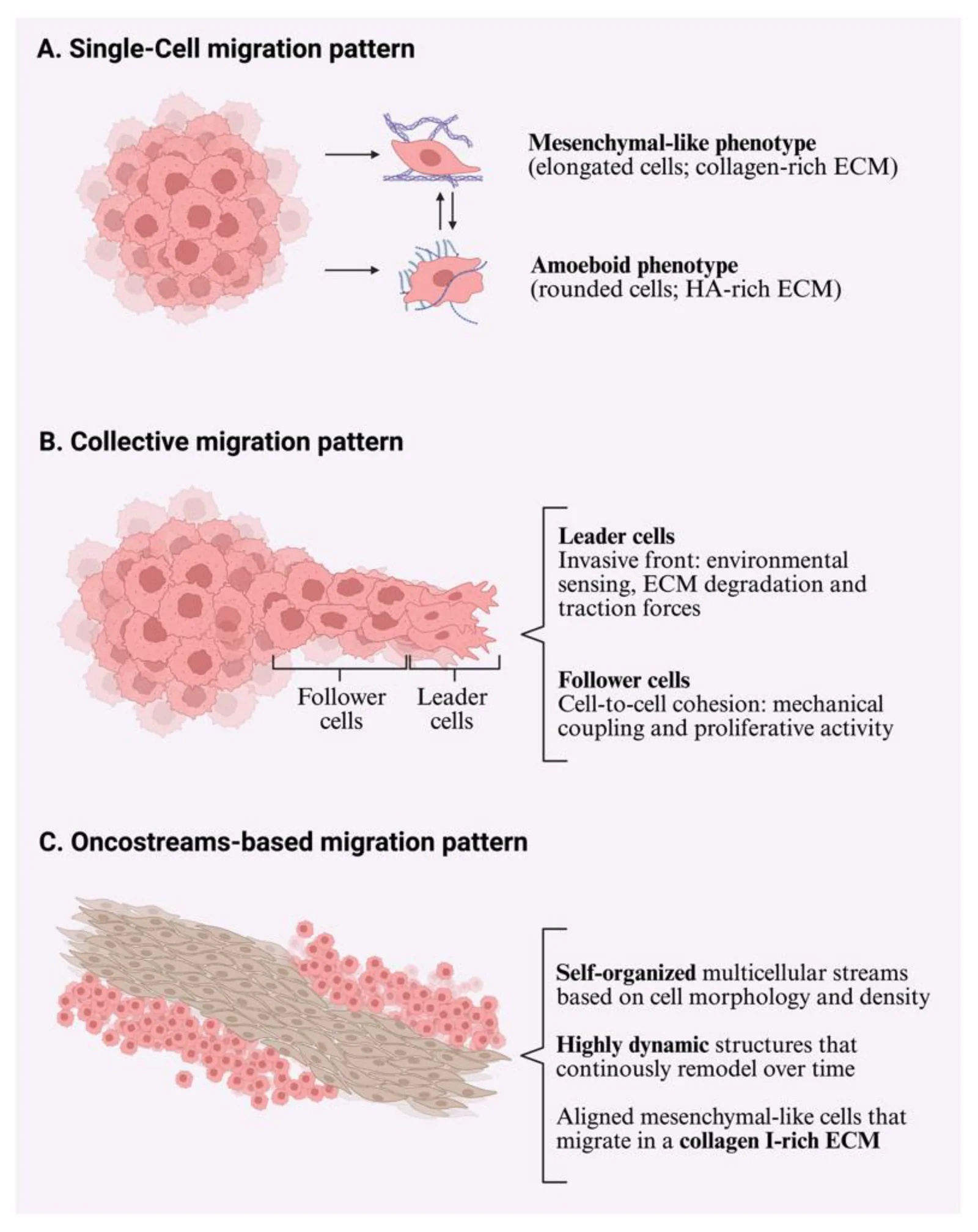
The figure summarizes the three distinct migration patterns described for GBM cells. (**A**) Single-cell migration pattern, including mesenchymal-like phenotype and amoeboid phenotype; (**B**) collective migration pattern, characterized by a hierarchical organization into leader and follower cells; and (**C**) oncostream-based migration pattern. Created in BioRender. Bucchieri, F. (2026) https://BioRender.com/ijsu7fs.

Historically, malignant glioma (including GBM) infiltration has been predominantly interpreted as a single-cell migratory process (Figure 1A), and, consequently, the mechanisms regulating individual cell migration have been extensively investigated [8,9,10].

Generally, cell migration is a highly coordinated process initiated by extracellular signals, including chemokines, cytokines, and growth factors, which induce cell polarization and establish a protrusive leading edge, oriented toward the migratory stimulus, and a contractile trailing edge. Actin polymerization at the leading edge, coupled with actomyosin contraction, generates the mechanical forces required for cell movement. These forces are then transmitted to ECM through transmembrane adhesion receptors, primarily integrins, enabling forward translocation of the cell body [11]. Tumor invasion further relies on the release of matrix metalloproteinases (MMPs), which degrade ECM components and facilitate the surrounding tissue infiltration [12]. Brain ECM is markedly different from that of most other tissues, as it is characterized by low stiffness and a scarcity of fibrillar proteins such as collagens, fibronectin, and laminin, except around blood vessels and the glia [13,14]. Instead, the brain parenchyma is predominantly composed of hyaluronic acid (HA) and other glycosaminoglycans (GAGs) associated with proteoglycans, creating a unique microenvironment that makes it difficult for invading cells to cross due to its low stiffness [13]. Consequently, rather than infiltrating the brain randomly, GBM cells preferentially migrate along pre-existing anatomical structures with higher mechanical stiffness, including white matter tracts, brain vasculature, and the pial surface [3,15,16]. Moreover, as tumor progression occurs, GBM cells actively remodel the surrounding ECM by degrading native components while secreting proteins that increase matrix stiffness, including fibronectin, laminin, and several collagen isoforms, thereby generating a microenvironment that progressively supports tumor invasion [17,18].

While GBM cells actively remodel the tumor microenvironment (TME) to support tumor growth and therapeutic resistance, they also encounter a highly heterogeneous non-neoplastic environment as they infiltrate into the healthy brain parenchyma. During migration, tumor cells interact with diverse resident cell populations, including astrocytes, microglia, neurons, and endothelial cells, as well as with ECM components, which provide biochemical and biomechanical cues affecting their migratory behavior. Therefore, GBM cells exhibit remarkable phenotypic plasticity and dynamically adapt their morphology and migratory strategy in response to the local microenvironmental cues [7,10].

The best-characterized single-cell invasion-associated program is the mesenchymal-like phenotype (Figure 1A). Although GBM cells do not undergo a canonical epithelial-to-mesenchymal transition (EMT) due to their non-epithelial origin, they can acquire a mesenchymal-like phenotype characterized by elongated morphology, enhanced cell-to-ECM interactions, proteolytic activity, and increased migratory capacity [19]. In GBM, mesenchymal protein expression is activated by several TME factors, including hypoxia [19,20,21], inflammation [22], and immune cells interaction [20,23,24], following the activation of multiple invasion-associated transcriptional regulators, including Nuclear Factor kappa-light-chain-enhancer of activated B cells (NF-κB), Twist family bHLH transcription factor 1 (TWIST1), Zinc Finger E-box-binding homeobox 2 (ZEB2), Wingless-related integration site (WNT)/β-catenin signaling pathway, Signal transducer and activator of transcription 3 (STAT3), CCAAT/enhancer-binding protein beta/delta (C/EBP-β/δ), Yes-associated protein/Transcriptional co-activator with PDZ-binding motif (YAP/TAZ) signalling pathways [20,21,22,25,26,27,28].

In response to environmental changes, GBM cells can reversibly switch from a mesenchymal-like to a rounded amoeboid phenotype (Figure 1A) [29,30,31], characterized by rapid individual migration, reduced dependence on proteolytic ECM remodeling, and increased reliance on Rho-ROCK-driven Myosin II activity to squeeze through pre-existing tissue spaces [32]. The transition from mesenchymal to amoeboid phenotype (MAT), and the opposite process (AMT), is tightly regulated by the balance between Ras-related C3 botulinum toxin substrate 1 (RAC1) and Rho/ROCK, which coordinate the cytoskeletal organization and cell contractility [33]. Inhibition of RAC1 often results in a MAT event [33,34], while, conversely, ROCK inhibition abolishes the amoeboid phenotype [33,34,35]. Importantly, MAT can be triggered by both microenvironmental changes and therapeutic interventions, highlighting the remarkable adaptability of GBM cells. For instance, migrastatic treatment with CCG-1423, which indirectly inhibits the cell adhesion protein CCN1, was ineffective in stopping GBM invasion due to MAT [30]. Similarly, treatment with MMP inhibitors was shown to promote the same process [35]. ECM composition and stiffness further influence the GBM cells’ migratory behavior. In vitro, a soft ECM, rich in HA, was shown to promote amoeboid migration of U87 cells, whereas a stiffer, collagen-rich environment favored the mesenchymal modality [36].

In summary, their remarkable plasticity allows GBM cells to maintain a significant migratory ability despite changes in the TME or therapeutic pressure, representing one of the major obstacles to effective anti-invasive therapies.

### 2.2. Collective Migration Pattern

Cancer cell dissemination has often been thought to occur primarily via a single-cell pattern, as tumor cells’ migratory ability increases when they lose cell-to-cell adhesion. However, substantial evidence indicates that tumor cells can metastasize cohesively as a group, sheet, or cluster [37]. Usually, collective migration is considered a hallmark of epithelial cancer invasion [38], whereas single cells preferentially migrate in a mesenchymal state. However, it has been reported that partial EMT, but not full EMT, a typical behavior also adopted by malignant gliomas, such as GBM, promotes collective migration (Figure 1B) [39,40].

Collectively migrating cells are not a group of independent cells that happen to move at the same speed and direction. Instead, they appear as an integrated multicellular system characterized by dynamic cell-to-cell adhesions and coordinated cytoskeletal organization, which enable force transmission and collective polarization, resulting in a more efficient migration toward a common direction and the acquisition of specific features [41]. Generally, this pattern of migration confers specific advantages, including increased resistance to detachment-induced cell death (anoikis), enhanced tolerance to shear stress, improved immune evasion, and greater metastatic potential [37].

Intercellular adhesion proteins, including tight junction (TJ) and adherens junction (AJ) proteins, are essential for maintaining the structural integrity and coordinated movement of collectively invading cells [42]. For instance, collectively invading malignant glioma cells showed an enrichment of the mesenchymal marker N-cadherin, along with the AJ proteins β-catenin and p120-catenin [43,44]. Conversely, disruption of intercellular adhesion through loss of the TJ protein CX43 (connexin-43) [45], or overexpression of the AJ destabilizer galectin-3 [44], promoted cluster disaggregation in high-grade gliomas. Beyond preserving multicellular cohesion, adhesion complexes also contribute to the establishment of collective polarity and to the functional organization of migrating cell groups. In fact, collective migration does not imply functional equivalence among cells. Instead, these groups are hierarchically organized into leader and follower cells [40]. Leader cells are located at the invasive front and execute a variety of migratory functions, including matrix degradation and path generation, whereas follower cells maintain tissue cohesion and proliferative capacity [41]. Specifically, mechanical studies showed that collective movement is primarily driven by traction forces generated by leader cells, whereas follower cells exert considerably smaller forces and are displaced through mechanical coupling within the cell group [41]. In brain tumors, the transmission of forces within the cellular mass is mainly mediated by AJ proteins, such as N-cadherin, which play a pivotal role in maintaining multicellular front-rear polarity during collective migration [46]. This process is partially coordinated by the N-cadherin ligand p120 through the regulation of RhoA and Rac1 signaling [43]. At a transcriptional level, GBM collective migration is further reinforced by the non-canonical NF-κB pathway. NF-κB-inducing kinase (NIK) expression was found to be enriched at the invasive front and promoted the collective invasion pattern by establishing a leader–follower phenotype and inducing the expression of MMPs and integrins [47]. Notably, despite their distinct roles, both leader and follower cells remain highly plastic and can dynamically interchange during the invasion process. In fact, in high-grade gliomas, both populations express N-cadherin through a YAP/TAZ-dependent pathway, although leader cells exhibit higher levels of surface and recycled N-cadherin, along with nuclear localization of YAP1, which is proposed to be determinant for their role [46].

However, a distinct leader–follower hierarchy has not been described in all forms of collective migration, suggesting that different programs may rely on alternative mechanisms of multicellular coordination [48,49]. For instance, a form of amoeboid-like collective migration was reported in colorectal cancer (CRC), where clusters of cancer cells were able to invade the surrounding tissue without the formation of adhesion-based protrusions [50]. Similarly to the MAT process, the inhibition of integrin-β1-based collective migration led to the switch towards an amoeboid-like phenotype in CRC and pancreatic cancer organoid models [51]. The amoeboid collective migration considers the cluster as a single cell, with a front-rear polarization, that moves through random contractile forces generated in the cluster and non-specific friction forces with the environment [52]. These examples provide evidence of collective migration modes that do not rely on the focal adhesion-based traction forces typical of leader cells. However, this phenomenon was reported primarily in CRC models, and whether it occurs in GBM remains to be determined.

### 2.3. Oncostream-Based Migration Pattern

The concept of collective migration has considerably expanded our understanding of GBM invasion by demonstrating that tumor cells can coordinate their movement through intercellular communication and mechanical coupling. However, recent evidence suggests that GBM cells are capable of a higher level of multicellular organization. Rather than simply migrating as cohesive groups, they can spontaneously assemble into highly ordered, dynamic structures, named oncostreams (Figure 1C) [53]. Oncostreams have been described in GBM as fascicles of elongated, spindle-shaped cells displaying mesenchymal-like properties that extend over 100–500 μm long and 50–200 μm wide, oriented toward a common direction [53]. Experimental studies have documented that oncostreams exhibit properties that resemble those of a nematic liquid crystal, a state where elongated cells act as rod-like units that preferentially align along a common axis, maintaining the ability to move collectively [54,55]. The structural alignment is further reinforced by N-cadherin overexpression, strengthening the evidence that these fascicles are composed of mesenchymal-like cells. Despite N-cadherin detection, no difference was found compared to the surrounding tissue [53], suggesting that N-cadherin expression is not a distinguishing feature of oncostreams.

Computational studies indicate that both cell morphology and cellular density act as key determinants of tumor self-organization. Accordingly, spindle-shaped cells are prone to align with neighboring cells to avoid collisions, establishing cell-to-cell interactions [56] supported by N-cadherin [53], thereby promoting the formation of stream-like architectures. In contrast, rounded cells lack this capacity to align and therefore remain in a more scattered conformation [56]. Cellular density has emerged as a strong driver in the emergence of such patterns, as it promotes the appearance of the nematic state [55,56]. Specifically, higher cell densities are predicted to promote the emergence of oncostream structures, including “streams” characterized by parallel fascicles of thin, slow-moving cells migrating in opposite directions, while reducing the occurrence of “flocks”, in which cells move collectively along the same direction. As glioma cell density varies both spatially and temporally within individual tumors and among patients [57], the local formation of oncostreams is likely to reflect highly aggressive tumor regions rather than a static histological feature.

Despite their highly ordered architecture, oncostreams remain dynamic structures that continuously adapt to changes in the local cellular environment. Computational modelling demonstrated that the presence of passive round cells with reduced migratory activity disrupted the self-organization process by interfering with cellular alignment and reducing the coherence of collective motion [58]. The loss of collective alignment is consistent with the liquid crystal model and contributes to the emergence of nematic topological defects, i.e., transient regions where the ordered arrangement of elongated cells is locally disrupted and subsequently reorganized [54]. Importantly, disruption of streams does not necessarily reduce tumor aggressiveness. Instead, passive cells appear to redirect collective behavior toward alternative organizational states [58]. Specifically, passive cells interfere with streams, whereas faster-moving flocks appear to segregate and exclude passive cells toward less organized regions, thereby preserving their collective structure. These findings suggest that streams may represent transient organizational states that progressively evolve into more stable flock arrangements.

An additional layer of complexity arises from findings showing that oncostreams are not purely tumor-derived structures, but rather multicellular assemblies that also incorporate cells of the TME, including tumor-associated microglia/macrophages (TAMs), which constitute a substantial proportion of the immune population in GBM [59]. Single-cell transcriptomic analyses have identified a distinct TAM subpopulation enriched within oncostream-associated invasive niches, characterized by ECM remodeling signatures and spatial alignment with invading GBM cells [60]. Moreover, it has been observed that TAM supports oncostreams’ stability through a Plexin-B2-mediated crosstalk, as inhibition of this gene impaired the directional migration of these cells, limiting tumor invasiveness [60,61].

Thus, oncostreams emerge as highly dynamic structures that continuously remodel in response to changes in cellular behavior and microenvironmental conditions.

Beyond cell morphology and density, another major determinant of oncostream formation is ECM composition. In fact, oncostreams display a distinct molecular signature enriched for ECM-related genes, such as collagen type-I chain A (COL1A1), ADAM metallopeptidase with thrombospondin type 1 motif 2 (ADAMTS2), and Matrix metallopeptidase 9 (MMP9). Among those, COL1A1 emerges as a key hub within this regulatory network [53]. As mentioned before, collagen is scarce in normal brain ECM [13,14], and GBM cells have shown the capacity to secrete collagen proteins to remodel their microenvironment [62]. Collagen deposition provides a mechanical scaffold that facilitates tumor invasion [17,18]. In this context, collagen I secretion promotes the alignment and collective migration of glioma cells, facilitating invasion, whereas its degradation disrupts oncostream organization in vitro [63] and reduces tumor invasiveness, prolonging survival in vivo [53].

Collagen I deposited within oncostreams is sensed through collagen-binding integrins, which mechanically couple the ECM to the actomyosin cytoskeleton. Consistent with their central role in cell-to-ECM interactions, aberrant integrin expression is widely associated with glioma invasiveness [64]. Accordingly, pharmacological inhibition of the collagen receptor α_2_β_1_ integrin disrupts oncostream formation in vitro [63]. Likewise, perturbation of the actomyosin machinery compromises oncostream integrity, as inhibition of actin polymerization with cytochalasin D or myosin II activity with blebbistatin disrupts collective organization and cell motility. Similarly, both pharmacological activation and inhibition of Rho GTPase signaling impair oncostream stability, indicating that maintenance of these structures depends on a finely tuned balance of Rho activity [63]. Upstream of these processes, intracellular Ca^2+^ signaling represents a major regulator of actomyosin dynamics by coordinating actin polymerization and myosin II activity through Ca^2+^-dependent pathways. Accordingly, perturbation of Ca^2+^ homeostasis profoundly affects cytoskeletal organization and glioma cell motility. For instance, increased intracellular Ca^2+^ levels enhance MMP2 expression and invasive capacity [65], whereas Ca^2+^ chelation suppresses glioma invasion [63].

Collectively, these findings indicate that oncostream formation relies on the coordinated integration of ECM remodeling, mechanotransduction, and cytoskeletal dynamics, suggesting that disruption of any component of this axis is sufficient to impair this particular invasion modality.

Oncostreams show substantial differences from the collective mesenchymal-like migration described broadly in GBM (Table 1). However, since it has been described only in recent years, our understanding of oncostream dynamics is still limited. It remains unclear whether oncostreams represent true multicellular collectives guided by cell-to-cell polarization, self-organized assemblies of individually migrating cells that align through local physical interactions [56], or an intermediate invasion state sharing features of both modes.

## 3. 3D In Vitro Models Recapitulating GBM Cell Invasion Properties

As outlined previously, GBM is characterized by extensive heterogeneity and diffuse infiltration of the surrounding healthy brain tissue, a hallmark that contributes substantially to poor prognosis. Elucidating the molecular and biophysical mechanisms underlying GBM cell migration and invasion is therefore essential for identifying novel therapeutic targets capable of limiting tumor dissemination and improving patient outcomes.

GBM invasion is primarily governed by two interconnected factors: the anatomical features of the surrounding tissue that provide permissive, low-resistance routes for migration, and the biochemical cues within the TME that regulate cell-to-matrix interactions, adhesion, and directional motility [66,67]. Recapitulating these features in vitro remains challenging, particularly in conventional 2D cultures. Instead, 3D models offer a more physiologically relevant approach to recreating the spatial and biochemical complexity of distinct tumor niches, including perivascular, hypoxic, and invasive microenvironments [68]. Importantly, GBM cell invasion is not governed by matrix stiffness alone, but rather by the interplay of distinct, although often interrelated, microenvironmental properties, including viscoelasticity and stress relaxation, matrix architecture and confinement, and time-dependent ECM remodeling, all of which can influence cellular migratory behavior.

Cancer cell invasion has traditionally been investigated using Matrigel, an Engelbreth–Holm–Swarm (EHS) mouse sarcoma-derived basement membrane (BM) preparation containing laminins, collagen IV, heparan sulfate proteoglycans, entactin/nidogen, and various growth factors. Its widespread use stems from its compositional similarity to the BM, an ECM niche that is rapidly infiltrated by tumor cells during invasion [69]. However, Matrigel presents several limitations for modeling GBM invasion. Its murine origin, poorly defined composition, substantial batch-to-batch variability, and collagen- and laminin-rich profile do not faithfully recapitulate the HA-rich ECM of the human brain [13].

In contrast, several biomaterials have recently been employed to mimic key features of the GBM ECM [70]. These include HA, the most abundant GAG in the brain ECM; collagen, an ECM component enriched in glioma tissue that is widely used because its concentration and organization can be tuned to modulate key physicochemical properties, such as stiffness and pore size; chitosan, a polysaccharide with structural similarities to GAGs; alginate, a hydrophilic polysaccharide that can undergo ionic crosslinking with calcium ions; and gelatin, a denatured collagen derivative that can be readily degraded by MMPs and hyaluronidases (HYALs) [71]. Moreover, these biomaterials are frequently combined to generate composite scaffolds with customizable biochemical and biophysical properties, thereby more closely recapitulating the complex GBM TME.

The following paragraphs describe the biomaterials more frequently used to create hydrogels able to more closely mimic the GBM ECM, whose complexity still represents a major challenge.

### 3.1. Collagen-Based Hydrogels

Collagen is an important structural component of the GBM ECM and can be deposited by tumor cells, contributing to the biochemical and mechanical properties of the TME and influencing tumor progression. To date, 29 collagen types have been identified and are broadly classified into three major groups: fibrillar, network-forming, and fibril-associated collagens. Among these, fibrillar collagens include types I, II, III, V, and XI, with collagen type-I the most widely used fibrillar collagen in biomaterial-based GBM models because of its structural and mechanical properties [72]. However, collagen extraction and purification often disrupt its native fibrillar architecture, resulting in hydrogels with mechanical properties that do not fully recapitulate those of the in vivo ECM. To overcome these limitations, collagen matrices can be chemically modified—for example, through crosslinking with EDC/NHS (1-ethyl-3(3-dimethylaminopropyl-carbodiimide hydrochloride/ N-hydroxysuccinimide) or genipin, or biologically modified through enzymatic crosslinking mediated by transglutaminase—to enhance their mechanical stability while preserving their biological functionality and cell-interactive properties [73,74]. However, it has been observed that crosslinking of collagen hydrogels increases their stability, but can decrease tumor cell migration speed. For instance, Kaphle et al. generated microtumors by encapsulating GBM cells within type-I collagen, followed by crosslinking of the collagen matrix with 8arm PEG Succinimidyl Glutarate (8S-StarPEG) [75]. This crosslinking significantly increased hydrogel viscosity, enhanced the resistance of the collagen matrix to collagenase-mediated digestion, and reduced tumor cell migration. In contrast, GBM cells cultured in non-crosslinked collagen hydrogels exhibited higher expression of matrix remodeling enzymes, including MMP-2, MMP-9, tissue plasminogen activator (tPA), and urokinase plasminogen activator (uPA), which was associated with increased matrix remodeling and cell migration speed [75].

A widely used collagen derivative for hydrogel fabrication is gelatin, which is obtained by the enzymatic denaturation of collagen and is readily degraded by MMPs and HYALs [71]. Among gelatin-based biomaterials, methacrylamide-functionalized gelatin (GelMA) has emerged as one of the most extensively employed platforms for GBM modeling owing to its excellent biocompatibility and highly tunable mechanical properties [76,77]. GelMA hydrogels have been shown to modulate tumor cell proliferation, gene expression, protein secretion, and therapeutic response, collectively promoting cellular phenotypes associated with GBM progression [77]. An additional advantage of GelMA hydrogels is their ability to support the formation of 3D vascular-like networks through the co-culture of endothelial cells and fibroblasts, thereby providing a more physiologically relevant model of the perivascular niche [78]. Importantly, the stiffness of GelMA hydrogels can be finely adjusted within the 1–10 kPa range, closely matching the elastic modulus reported for human GBM tissues [79]. Using this platform, Ngo et al. identified nine proteins secreted by self-assembled endothelial networks that are closely associated with GBM biology. Several of these proteins localize to the perivascular regions of GBM and have been implicated in promoting tumor cell proliferation, invasion, and other processes involved in tumor progression [77]. These findings highlight the versatility of GelMA hydrogels for recreating both the mechanical and cellular complexity of the GBM TME, making them a valuable platform for investigating tumor–stroma interactions.

Collagen and collagen-derived biomaterials can also serve as valuable platforms for investigating the crosstalk between immune cell populations within the TME and its contribution to GBM progression. Studies have shown that infiltrating and resident immune cells, particularly microglia, can account for up to one-third of the solid tumor mass [80]. Tissue engineering platforms have been employed to elucidate the interactions between patient-derived GBM cells and microglia. For instance, studies using GelMA hydrogels demonstrated that the presence of patient-derived GBM cells induced morphological changes in microglia consistent with an activated phenotype. Notably, microglial co-culture significantly reduced GBM cell invasion while simultaneously promoting tumor cell proliferation, highlighting the complex and context-dependent role of microglia in GBM progression [81]. The reciprocal effects of GBM cells on microglial behavior have also been investigated using a type-I collagen hydrogel derived from rat tail and incorporated into a micro-engineered multi-channel microfluidic device. In this model, the presence of GBM cells significantly enhanced microglial invasion into the collagen matrix. Interestingly, this increase in microglial invasion was primarily driven by cellular contractility and occurred independently of both MMP activity and cellular glycolysis [82].

### 3.2. HA-Based Hydrogels

HA-based hydrogels represent a compromise between biological relevance, reproducibility, and tunability. Their relatively well-defined composition enables controlled modulation of biochemical and biophysical properties, including stiffness, degradability, and ligand presentation, facilitating the systematic investigation of how specific ECM cues influence GBM cell behavior [83]. In addition to its structural and mechanical roles, HA serves a series of biological functions, from engaging with cell surface receptors such as CD44 to undergoing remodeling processes via cell surface enzymes capable of HA synthesis, i.e., the hyaluronan synthases, and HA degradation, i.e., HYAL [84]. GBM is strongly characterized by invasion into the HA-rich brain parenchyma through the “leader-follower” mechanism, in which leader cells locally digest the matrix with HYAL, generating microscale channels that facilitate the rapid invasion of follower cells [85]. The importance of HA binding and remodeling proteins in GBM progression has prompted the development of HA-based matrix platforms to investigate GBM invasion in vitro. However, while HA in the brain consists primarily of high-molecular-weight (HMW, ≈1.5 MDa) chains that are noncovalently crosslinked with large tenascin proteins decorated with lectins, HA hydrogels are typically fabricated in vitro by chemically modifying the backbone of low-molecular-weight (LMW, ≈60 kDa) HA chains. This is partly because the lower viscosity of LMW HA solutions facilitates their synthesis, assembly, and use in cell culture, while allowing the introduction of functional groups that support the fast and efficient conjugation of crosslinkers. The crosslinking chemistries traditionally used in HA hydrogels are based on static covalent bonds that allow the matrix to store, but not dissipate, the applied stresses [86,87]. Nevertheless, HMW HA formulations may provide access to a broader range of viscoelastic properties, as the longer polymer chains themselves can contribute to stress relaxation through increased chain entanglement, deformation, and rearrangement. In a recent study, Carvalho EM et al. leveraged the ability of HMW polymer entanglements to rearrange and reorganize to develop and characterize a series of HA-based hydrogels with different degrees of stress relaxation. They found that the most stress-relaxing formulation exhibited stress-relaxation properties comparable to those of brain tissue. The authors also found that increasing HA stress relaxation promoted faster GBM cell invasion, which occurred through the “leader-follower” mechanism reminiscent of GBM invasion in vivo [83]. Moreover, the use of HMW HA-based scaffolds, which more closely recapitulate the composition of the GBM ECM in vivo, may be advantageous because both the molecular weight and the degree of chemical modification of HA can profoundly influence its biological activity, including CD44 receptor activation [88]. An additional consideration is the dynamic turnover of HA during 3D culture. HYAL-mediated degradation can generate HA fragments of different molecular weights, which may elicit biological responses distinct from those associated with HMW HA and modulate CD44-mediated signaling. However, conventional synthetic HA hydrogels may not fully reproduce the continuous conversion of HMW HA into lower-molecular-weight fragments occurring in the native tumor microenvironment, particularly when chemical modification and crosslinking alter the accessibility of HA to enzymatic degradation. Consequently, changes in HA molecular weight during culture may influence CD44 engagement and cell–matrix interactions, potentially affecting the interpretation of mechanotransduction and GBM invasion studies. These considerations highlight the importance of accounting for both the initial molecular weight of HA and its time-dependent enzymatic remodeling when designing and interpreting HA-based models [83].

**Figure 2:**
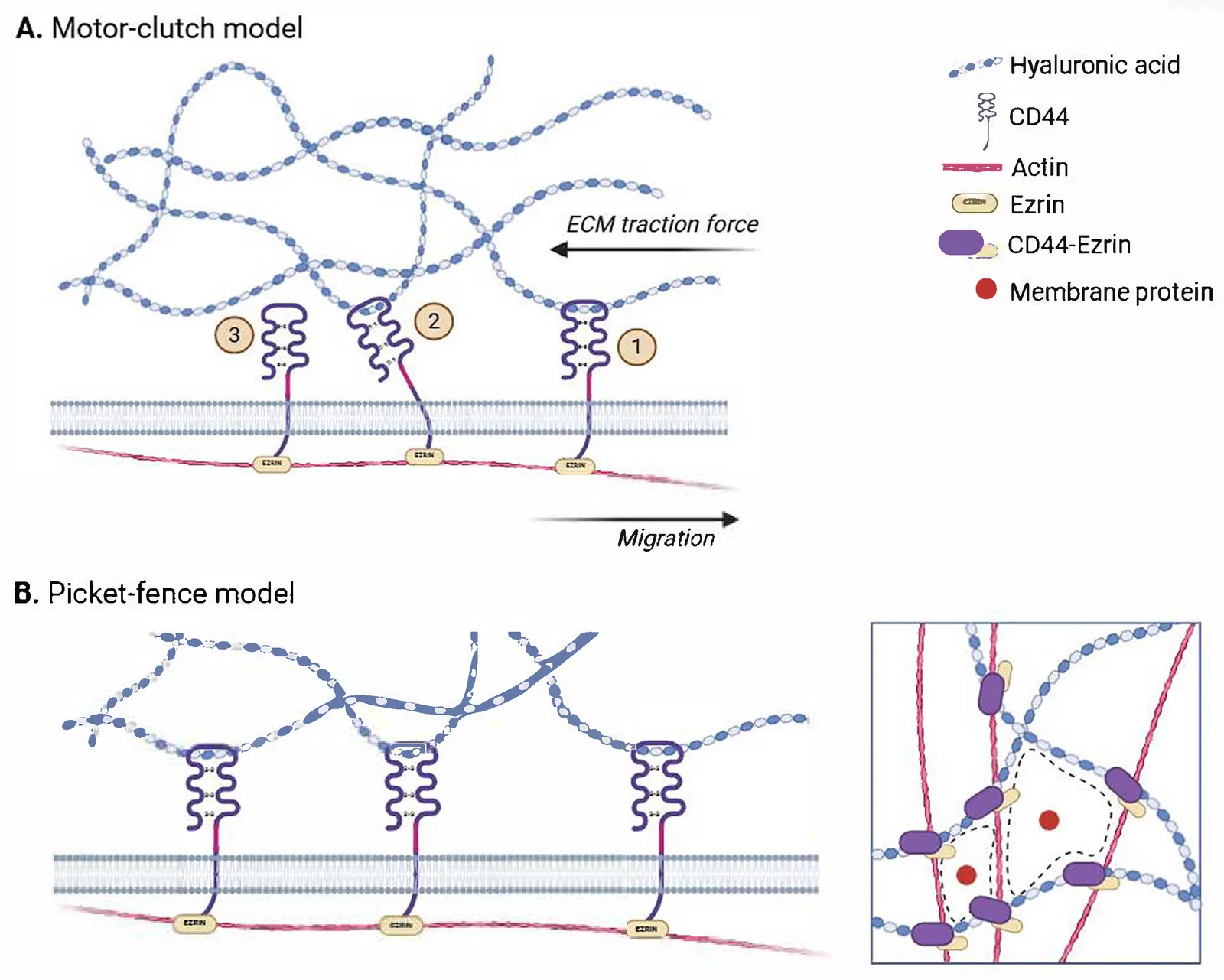
Schematic representation of two models that explain the HA-CD44 interactions. (**A**) In the motor-clutch model, ECM traction forces support GBM cell migration. CD44 binds the glycocalyx component hyaluronic acid via its extracellular domain. Intracellularly, it engages with the cortical actin cytoskeleton via ezrin and other linker proteins. CD44 (“clutch”), once engaged (1), undergoes a load (2) and fail (3) cycle. (**B**) In the picket-fence model, CD44 (“picket”), bound to HA, and cortical actin (“fence”) demarcate membrane domains (“corrals”, black dotted lines) that regulate diffusion of membrane remodeling GBM proteins (red dots). Created in BioRender. Bucchieri, F. (2026) https://BioRender.com/ijsu7fs.

The presence of CD44 in experimental models is relevant for investigating therapeutic strategies aimed at preventing GBM cell invasion. CD44 can function as a mechanosensor, mediating the effects of substrate stiffness on cell proliferation and motility [89]. Several studies suggested that CD44-mediated cell behavior may be explained by both the “motor-clutch” and “picket-fence” models. According to the “motor-clutch” model, effective cell migration depends on the mechanical coupling of ECM-bound receptors to the actin cytoskeleton through intracellular adaptor proteins [90]. In the case of CD44, this connection is mediated by the ezrin adaptor protein, which, upon phosphorylation and transition to its “open” conformation, links the intracellular domain of CD44 to actin filaments, allowing the transmission of traction forces that support cell migration [91] (Figure 2A). In contrast, the “picket-fence” model describes CD44 as a structural regulator of plasma membrane organization. In this framework, cortical actin filaments constitute the “fences”, while CD44 molecules, tethered to actin via ezrin, act as the “pickets.” These membrane-associated pickets create localized membrane “corrals” that restrict the lateral diffusion of membrane proteins, including integrins and CD44. Consequently, increased CD44 tethering reduces membrane diffusion and receptor clustering, thereby limiting localized increases in membrane tension (Figure 2B). This spatial organization is essential for several polarization-dependent cellular processes, such as chemotaxis and phagocytosis, and is likely to play an important role in regulating GBM cell migration and invasion [91,92]. A promising strategy to prevent GBM cell invasion involves inhibiting ezrin activation using NSC668394. However, in very soft tissue microenvironments, where cells interact with the extracellular matrix through integrins rather than CD44, low doses of NSC668394 may trigger a phenotypic switch from mesenchymal-like to amoeboid-like migration in GBM cells, potentially contributing to therapeutic resistance [91].

This study demonstrates that GBM cell invasion exhibits a biphasic dependence on HA concentration, supporting the “motor–clutch” model of cell migration, in which CD44–ezrin complexes function as molecular “clutches” that regulate migratory activity independently of integrin-mediated adhesion. Furthermore, the findings support previous evidence that CD44–ezrin complexes act as molecular “pickets” within the anchored “picket-fence” model. According to this model, cell migration depends on the dynamic spatiotemporal coordination of picket anchoring and release, regulated by ezrin phosphorylation and dephosphorylation [91].

These findings confirm, as discussed in the previous section, that low cell–matrix adhesion and confined environments, such as matrix pores, favor the MAT.

HA-based hydrogels have also been employed to investigate the role of glutamate signaling in 3D GBM invasion [93]. Glutamate, the primary excitatory neurotransmitter in the CNS, is present at elevated levels in the GBM TME, where it promotes proliferation, migration, and therapeutic resistance, while inducing excitotoxic damage in neighboring neurons [94]. Using HA-based hydrogels, it has been shown that pharmacological inhibition of the N-methyl-D-aspartate receptor (NMDAR), a glutamate receptor, significantly reduces invasion from GBM tumorspheres. This result has been consistently observed across several established GBM cell lines, as well as in patient-derived xenograft models [93].

A major advantage of HA-based hydrogels over other matrices, such as collagen I and Matrigel, has emerged from studies investigating transforming growth factor-β (TGF-β)-driven glioma stem cell (GSC) invasion [95]. In HA-based hydrogels, GSCs exhibit distinct invasive behaviors that correlate with TGF-β receptor 2 expression and SMAD2 phosphorylation, indicating that the response to TGF-β can be predicted by the activation status of this signaling pathway. Notably, TGF-β-induced invasion is strongly dependent on the presence of RGD adhesion peptides conjugated to the HA backbone, whereas MMP-mediated degradability has little or no influence on the invasive response. These findings are consistent with the established role of TGF-β/SMAD signaling in promoting tumor microtube formation and GBM invasion. Importantly, HA-based hydrogels provide a low-invasive baseline in the absence of TGF-β stimulation, allowing the specific contribution of this signaling pathway to be accurately evaluated [95]. This feature contrasts with collagen I and Matrigel, which inherently support robust cell invasion even without exogenous cytokine stimulation because of their fibrillar architecture, micrometer-scale pore size, and the presence of endogenous biochemical and topographical pro-migratory cues. In comparison, the nanometer-scale pore structure and chemically defined composition of HA-based hydrogels minimize these confounding factors, enabling a more controlled analysis of invasion mechanisms [96,97]. Furthermore, the high degree of tunability of HA hydrogels allows individual matrix parameters, such as RGD ligand density, to be systematically modified, demonstrating that integrin-mediated adhesion is required for TGF-β-induced GSCs invasion [95].

Another distinctive feature of HA-based hydrogels has emerged from studies investigating the effect of cofilin-1 (CFL1) suppression on GBM cell migration. In HA-based 3D cultures, CFL1 knockout cells displayed a unique migratory phenotype characterized by the formation of a greater number of markedly elongated protrusions compared to control cells. Interestingly, this effect appeared to be specific to the HA microenvironment, as CFL1 depletion did not enhance cell motility in 3D collagen matrices or in conventional Transwell migration assays [98]. Within HA-based 3D matrices, suppression of CFL1 promoted the extension of filopodia and microtentacle-like protrusions. Tran VD et al. proposed that the loss of CFL1 enhanced the stabilization of actin filaments within these protrusive structures, thereby facilitating the ability of GBM cells to penetrate the HA-rich matrix [98]. These findings highlight how HA-based hydrogels can reveal migration mechanisms that are not readily detectable in other experimental platforms, underscoring the importance of matrix composition in regulating cytoskeletal dynamics and invasive behavior.

### 3.3. Collagen–HA-Based Hydrogels

Collagen and HA are frequently combined in hydrogel-based models of GBM to recapitulate key features of the TME. Their complementary properties allow the generation of viscoelastic and fibrillar matrices that provide both structural support and dynamic cues relevant to 3D cell culture. The interplay between ECM composition and mechanics is a critical determinant of GBM cell behavior, raising the question of how tumor-derived ECM components actively remodel the surrounding matrix to promote invasion. A recent study employed an engineered 3D HA-based ECM platform to investigate the early ECM remodeling events associated with GBM invasion. By combining metabolic labeling of secreted proteins with unbiased matrisome (an ensemble of ECM proteins and ECM-associated factors that collectively contribute to the assembly, organization, and remodeling of the ECM [99]) analysis and targeted candidate-based approaches, the authors identified collagen VI as a tumor-derived mediator of invasion [100]. Specifically, collagen VI deposition progressively stiffened the initially compliant HA matrix, thereby activating mechanotransductive signaling through specific integrin subtypes. This, in turn, promoted β-catenin activation and upregulated the mesenchymal transcription factor ZEB1, ultimately enhancing the invasive behavior of GBM cells. Notably, collagen VI secretion was markedly increased in a model of bevacizumab-resistant GBM, whereas its suppression significantly attenuated hypoxia-induced invasion and reduced the expression of mesenchymal markers [100].

A collagen–HA-based hydrogel has also been employed to recapitulate the invasive GBM TME by incorporating human astrocytes, microglia, and patient-derived GSCs [101]. Invasive tumor border regions are characterized by pressure gradients generated by densely populated tumor regions and leaky vasculature. In the model developed by Howerton et al., elevated interstitial fluid flow can be mimicked by applying a fluid pressure head across the hydrogel. Moreover, the model can be tuned to reproduce inter- or intra-patient variability in cellular ratios, flow rates, or matrix stiffnesses. This platform enables quantitative assessment of GSC invasion under physiologically relevant microenvironmental conditions [101].

Considering collagen-derived materials, promising HA gelatin-based hydrogels have also been developed. When gelatin is functionalized with methacrylamide groups, these reactive sites enable the covalent immobilization of HA within the hydrogel network. A few years ago, an HA-decorated GelMA hydrogel was employed to investigate the effects of matrix stiffness and HA presentation on GBM invasion. Interestingly, GBM invasion was enhanced in softer hydrogels but reduced in the presence of matrix-bound HA [102]. Additionally, matrix-bound HA has been shown to play an active role in the efficacy of targeted therapies. In a study investigating the impact of brain-mimetic HA content on the response of patient-derived GBM cells to erlotinib, the inclusion of brain-mimetic HA attenuated the effects of repeated erlotinib exposure, with prolonged treatment failing to significantly reduce GBM cell proliferation or migration [103]. Given that HA levels within the TME are continuously modulated through cell-mediated HA remodeling and synthesis, these findings highlight the need for further studies to elucidate the specific contribution of extracellular HA to therapeutic efficacy, including the relative roles of matrix-bound versus soluble HA, molecular weight, and dynamic remodeling.

Importantly, although matrix stiffness is frequently used to characterize these composite hydrogels, it does not fully capture their mechanical behavior. Viscoelasticity and stress-relaxation kinetics may also influence GBM cell–matrix interactions and invasive behavior and should therefore be considered alongside elastic stiffness when designing and interpreting these models. An overview of the main characteristics of the 3D in vitro hydrogel-based models discussed in this section is provided in Table 2.

**Table 2:** Summary of the main characteristics of 3D in vitro hydrogel-based models described in this section.

Hydrogel Composition	Biochemical and Mechanical Characteristics	Cellular Source	Modeled Microenvironmental Feature	Inferred Migration/Invasion Phenotype	Experimental Readout	Limitations
Type-I collagen crosslinked with 8S-StarPEG	Increased viscosity and resistance to digestion	U87 glioma cells	Increased matrix remodeling	Collective migration pattern	Cell migration distance and speed	Cells cannot represent the highly invasive behavior of primary glioma cells [75]
Methacrylamide-functionalized gelatin (GelMA)	Highly tunable stiffness, elasticity and degradation via crosslinking density	U87-MG cells + endothelial cells (HUVECs) + fibroblasts (NHLFs) Patient-derived GBM cells + HMC3 microglia cells	Formation of 3D vascular-like networks Microglial co-culture significantly reduced GBM cell invasion while simultaneously promoting tumor cell proliferation	Single-cell migration pattern (ameboid phenotype) Single-cell migration pattern (ameboid phenotype)	Single-cell morphology (rounded cell shape and membrane blebbing) and migration dynamics (single-cell trajectories and migration speed) Microglial morphology (rounded amoeboid shape) and 3D spheroid invasion assay, quantified as normalized invasion distance (average invasion-front radius relative to the initial spheroid radius)	Model vascular cell types may behave differently from brain-derived sources [79] The study examines processes linked to GBM progression using patient-derived GBM specimens in response to microglia, but GBM contain a heterogeneous mix of cells [81]
Hyaluronic Acid (HA)	Non-fibrillar matrix with tunable stiffness and crosslinking density	Patient-derived GBM cells Patient-derived glioma stem cells (GSCs)	Generation of microscale channels via HYAL-mediated matrix digestion Tumor microtube formation	Spheroid-associated collective migration pattern Spheroid-associated collective migration pattern	3D spheroid invasion, quantified by measuring cell invasion distance from the spheroid 3D spheroid invasion index, quantified by the inverse circularity of the spheroid invasion front, with qualitative assessment of multicellular protrusions and escaped cells.	Incomplete recapitulation of the architecture, viscoelasticity, and heterogeneity of the TME [91,95,98]
Collagen–HA	Viscoelastic and fibrillar matrix that provide both structural support and dynamic cues	Patient-derived GSCs, human astrocytes and microglia	Elevated interstitial fluid flow can be mimicked by applying a fluid pressure head across the hydrogel	Single-cell migration pattern (mesenchymal phenotype)	Percentage of glioma stem cells (GSCs) invading through the hydrogel	This model cannot yet replicate complex tissue architecture like vasculature or neuronal patterning [101]
GelMA-HA	Tunable mechanical properties, degradability, and porosity	Patient-derived GBM cells	Immobilization of HA within the hydrogel network	Collective migration pattern	3D invasion, quantified using a dextran-bead assay, cell migration distance and spatial distribution	Limited recapitulation of the native brain ECM architecture and viscoelasticity [102,103]

### 3.4. Limitations and Future Development of Hydrogel-Based Glioma Invasion Models

Despite their advantages in recapitulating selected features of the GBM microenvironment, hydrogel-based models present several limitations that should be considered when interpreting invasion-related findings. First, individual biomaterials reproduce only specific aspects of the native brain ECM: Matrigel is limited by its murine origin, poorly defined composition, and batch-to-batch variability [70], whereas collagen-based hydrogels may exhibit fibrillar architectures and mechanical properties that differ from those of brain tissue [72]. HA-based hydrogels offer greater compositional control but often rely on chemically modified, low-molecular-weight HA and static covalent crosslinking, which may not fully recapitulate the molecular organization, viscoelasticity, and dynamic remodeling of native brain ECM [83]. Similarly, composite hydrogels, such as GelMA–HA, provide tunable biochemical and mechanical properties, but the simultaneous effects of matrix composition, ligand density, stiffness, and crosslinking can complicate the identification of the specific cues driving GBM invasion [103]. Furthermore, most hydrogel-based systems incompletely reproduce the structural heterogeneity of brain tissue, including its anatomical migration routes, cellular diversity, and spatially heterogeneous biochemical and mechanical cues.

Importantly, the mechanical characterization of hydrogel-based GBM models should extend beyond static elastic stiffness to account for the time-dependent viscoelastic behavior of the matrix. Although Young’s modulus quantifies resistance to elastic deformation, stress relaxation describes how rapidly the stress dissipates under sustained deformation and may therefore influence how cells generate traction forces and remodel their surroundings during migration. In GelMA- and HA-based networks, crosslinking density and chemical functionalization can affect both elastic stiffness and stress-relaxation kinetics; however, these parameters are not necessarily coupled, and their relationship depends on the network architecture and crosslinking chemistry. Accordingly, the effects of matrix mechanics on GBM invasion cannot be inferred from stiffness measurements alone. This distinction is particularly relevant to leader–follower invasion, in which local matrix degradation and reorganization by leader cells may create permissive migration paths for follower cells. More broadly, the interplay between elastic resistance, stress dissipation, matrix architecture, and degradability may influence cell–matrix adhesion and cytoskeletal dynamics, thereby modulating migratory behavior and potentially contributing to transitions between mesenchymal-like and amoeboid-like states.

Future hydrogel designs should therefore aim to characterize stiffness and stress-relaxation kinetics separately and, where feasible, independently tune these properties to establish their respective contributions to GBM invasion.

An additional critical consideration is the cellular source used to establish these models, as established cell lines, patient-derived GBM cells, and GSCs may differ in their molecular profiles, receptor expression, and intrinsic migratory properties. Consequently, model selection may influence the migration phenotype observed, cellular responsiveness to matrix-derived biochemical and mechanical cues, and the apparent efficacy of therapeutic interventions, potentially limiting the generalizability of findings across experimental systems and patient populations. Although more complex models can incorporate vascular and immune components, reproducing the dynamic interactions among tumor cells, stromal and immune populations, and the surrounding neural tissue remains challenging. Finally, differences in biomaterial composition, fabrication methods, cellular sources, and experimental conditions can limit reproducibility and hinder direct comparisons across studies. Therefore, hydrogel-based models should be regarded as complementary platforms whose suitability depends on the specific mechanisms under investigation, rather than as comprehensive representations of the native GBM microenvironment. In this context, future developments should focus on designing biomaterials that more closely reproduce the biochemical composition, viscoelasticity, and dynamic remodeling of native brain ECM, while enabling the independent control of relevant parameters, including matrix stiffness, ligand density, pore architecture, and degradability. In particular, incorporating HMW HA and dynamic crosslinking strategies may improve the representation of native HA organization and stress-relaxation behavior. Integrating these biomaterials into more complex platforms, including microfluidic systems, vascularized models, and multicellular cultures incorporating patient-derived GBM cells, astrocytes, microglia, and endothelial cells, could further improve the representation of tumor–microenvironment interactions and spatially heterogeneous invasion patterns. Importantly, the use of multiple patient-derived models representing distinct molecular and phenotypic characteristics, including different migratory states, will be essential to determine whether observed invasion patterns, matrix responses, and treatment effects reflect generalizable biological mechanisms or model-specific behaviors. Combining these approaches with standardized characterization methods, quantitative live-cell imaging, and patient-derived experimental systems will be essential to improve reproducibility, facilitate comparisons across studies, and establish more reliable platforms for investigating invasion mechanisms and evaluating therapeutic strategies.

## 4. Conclusions

GBM represents one of the most significant clinical and therapeutic challenges in neuro-oncology. Standard treatment consists of maximal safe surgical resection followed by concomitant radiotherapy and chemotherapy with temozolomide (TMZ), and adjuvant post-radiation treatment consisting of TMZ alone [104]. This regimen is often personalized according to the patient’s age, tolerance and pre-existing medical conditions. However, this combinatorial therapy does not comprehensively clear all tumor cells, which can escape complete surgical resection, inducing recurrence [3]. Accumulating evidence indicates that brain parenchyma infiltration by GBM cells is not driven by a single, stereotyped migration program, but can occur through distinct migration patterns, ranging from the migration of individual cells to collective migration. In this context, invasive GBM cells show remarkable phenotypic plasticity in dynamically modulating their migratory behavior in response to local microenvironment signals [7,11]. One of the most complex patterns is based on the formation of higher-order, self-organizing multicellular structures called “oncostreams”, which display dynamic properties typical of nematic liquid crystals and are associated with rapid and aggressive infiltration [53,54,55]. Rather than being passive assemblies of cells, oncostreams actively remodel the surrounding ECM by the deposition of COL1A1 and matrix-degrading proteases to form stiff and aligned tracks that guide and sustain their coordinated progression [53,63]. However, it remains an open question whether oncostreams represent a biologically driven collective migration mode regulated by stable intercellular adhesions, or an emergent physical phenomenon in which cells align due to high packing density and steric constraints [56,63]. Therefore, elucidating these complex mechanisms is essential to identify new therapeutic strategies to limit tumor dissemination and improve patient outcomes.

Reproducing these phenomena in vitro requires moving from oversimplified 2D cultures, which fail to reproduce the spatial organization and cellular crosstalk, toward 3D biomimetic experimental platforms capable of combining reproducibility with physiological relevance [68,71]. One of the main challenges is to faithfully recapitulate the distinctive biochemical and biophysical properties of the brain ECM, since, unlike other tissues, it is characterized by a soft and highly hydrated environment enriched in HA and PGs, while it lacks fibrillar proteins such as collagen [13,14,83]. Traditional Matrigel, a mouse sarcoma-derived BM preparation, possesses a complex composition with a relatively high content of basement-membrane proteins that differ substantially from the HA-rich ECM of the brain, thereby failing to reproduce its viscoelastic characteristics, particularly time-dependent stress relaxation, which can dynamically regulate GBM invasion [83]. To overcome these limitations, recent efforts in tissue engineering have focused on biomimetic 3D hydrogels [69,70]. In particular, recent progress in biomaterials has focused on composite matrices, such as collagen–HA and GelMA-HA hydrogels, designed to combine key features of the physiological brain ECM with those of the remodeled TME, integrating the native viscoelasticity of HA with fibrillar networks [100,102]. These composite scaffolds have multiple biochemical and biophysical properties that can be precisely adjusted, enabling the controlled modulation of matrix composition, stiffness, viscoelasticity, and ligand presentation to more closely recapitulate key features of the GBM TME. From a translational perspective, hydrogel-based 3D models should be distinguished from biomaterials specifically designed as therapeutic platforms. In the former, hydrogels primarily serve as experimental tools to investigate GBM invasion, test mechanistic hypotheses, evaluate candidate drugs, and potentially support patient-specific modeling. By contrast, emerging migration-guiding or migration-trapping biomaterials aim to actively redirect or constrain tumor cell movement and therefore represent a distinct, prospective therapeutic strategy. Although increasingly biomimetic models may improve the relevance of preclinical investigations, their greater complexity has not yet been shown to translate into improved clinical outcomes. Their current translational value therefore lies primarily in mechanistic discovery and preclinical evaluation, while their contribution to patient stratification and therapeutic development remains to be established. Moreover, these systems allow us to understand how defined matrix properties affect the emergence and maintenance of distinct migration strategies, providing a valuable tool to investigate the mechanisms underlying GBM invasion.

## Figures and Tables

**Table 1:** Summary table showcasing differences and similarities between collective migration pattern of high-grade gliomas and oncostreams.

	Collective Migration	Oncostreams
**Cell morphology**	Mesenchymal-like cells with front-rear polarity and leading-edge protrusions	Spindle-shaped, elongated cells with mesenchymal proprieties
**Cellular organization**	Polarized groups, chains or networks of cells. Functional leader–follower distinction	Fascicles of aligned cells. The streams show polarization, but front-rear hierarchy is not demonstrated
**Cellular cohesion**	AJ proteins (N-cadherin, p120) connect cells and allow force transmission	Cells cohesion depends on physical proprieties (density and morphology) and mechanical interaction (N-cadherin)
**Extracellular matrix**	ECM remodeling support collective invasion, cells may exploit pre-existing structures	Enriched in Collagen-I fibers aligned along cells fascicles
**Direction of movement**	Frontal leading edge controls advancement in response to biochemical gradients	Spontaneous alignment defines a common axis, direction is influenced by segregation of other streams and non-migrating cells
**Identification**	Immunostaining and live-cell imaging are required for identification	Histological identification by H&E allows direct visualization
**Molecular signature**	Overexpression of NIK, MMPs, integrins define leader cells, YAP/TAZ differential expression between leader-follower	Overexpression of COL1A1, MMP9, ADAMTS2, ACTA2

## Data Availability

No new data were generated or analyzed in this review. The information discussed in this article was obtained from previously published studies identified through literature searches in PubMed and Google Scholar, as described in “Literature Search Strategy Section”.

## Abbreviations

The following abbreviations are used in this manuscript:

3D	Three-Dimensional
8S-StarPEG	8arm PEG Succinimidyl Glutarate
ADAMTS2	ADAM Metallopeptidase with Thrombospondin type 1 motif 2
AJ	Adherens Junction
AMT	Transition from Ameboid to Mesenchymal phenotype
BM	Basement Membrane
C/EBP-β/δ	CCAAT/Enhancer-Binding Protein beta/delta
CFL1	Cofilin-1
COL1A1	Collagen type-I chain A
CNS	Central Nervous System
CRC	Colorectal Cancer
ECM	Extracellular Matrix
EHS	Engelbreth–Holm–Swarm
EMT	Epithelial-to-Mesenchymal Transition
GAG	Glycosaminoglycan
GBM	Glioblastoma
GelMA	Methacrylamide-functionalized Gelatin
GSCs	Glioma Stem Cells
HA	Hyaluronic Acid
HMW	High-Molecular-Weight
HYALs	Hyaluronidases
LMW	Low-Molecular-Weight
MAT	Transition from Mesenchymal to Ameboid phenotype
MMP9	Matrix Metallopeptidase 9
MMPs	Matrix Metalloproteinases
NF-κB	Nuclear Factor kappa-light-chain-enhancer of activated B cells
NIK	NF-κB-Inducing Kinase
NMDAR	N-Methyl-D-Aspartate Receptor
PG	Proteoglycan
RAC1	Ras-related C3 botulinum toxin substrate 1
STAT3	Signal Transducer and Activator of Transcription 3
TAMs	Tumor-associated Microglia/macrophages
TGF-β	Transforming Growth Factor-β
TJ	Tight Junction
TME	Tumor Microenvironment
TMZ	Temozolomide
TWIST1	Twist family bHLH transcription factor 1
YAP/TAZ	Yes-Associated Protein/Transcriptional co-activator with PDZ-binding motif
WNT	Wingless-related integration site
ZEB2	Zinc Finger E-Box-binding homeobox 2
